# *O*-GlcNAcylation: A Sweet Hub in the Regulation of Glucose Metabolism in Health and Disease

**DOI:** 10.3389/fendo.2022.873513

**Published:** 2022-04-22

**Authors:** Maria J. Gonzalez-Rellan, Marcos F. Fondevila, Carlos Dieguez, Ruben Nogueiras

**Affiliations:** ^1^ Department of Physiology, CIMUS, University of Santiago de Compostela-Instituto de Investigación Sanitaria, Santiago de Compostela, Spain; ^2^ CIBER Fisiopatología de la Obesidad y Nutrición (CIBEROBN), Instituto de Salud Carlos III, Madrid, Spain

**Keywords:** O-GlcNAcylation, glucose, diabetes, insulin resistance, glucose homeostasis

## Abstract

*O*-GlcNAcylation is a posttranslational modification ruled by the activity of a single pair of enzymes, *O*-GlcNAc transferase (OGT) and *O*-GlcNAcase (OGA). These two enzymes carry out the dynamic cycling of *O*-GlcNAcylation on a wide range of cytosolic, nuclear, and mitochondrial proteins in a nutrient- and stress-responsive manner. To maintain proper glucose homeostasis, a precise mechanism to sense blood glucose levels is required, to adapt cell physiology to fluctuations in nutrient intake to maintain glycemia within a narrow range. Disruptions in glucose homeostasis generates metabolic syndrome and type 2 diabetes. In this review we will discuss and summarize emerging findings that points *O*-GlcNAcylation as a hub in the control of systemic glucose homeostasis, and its involvement in the generation of insulin resistance and type 2 diabetes.

## Introduction

Glucose homeostasis is essential for life since glucose is the main source of energy for most eukaryotic cells. In mammals, glucose is the principal source of energy for “glucodependent” tissues such as brain and blood cells, so its blood levels are maintained within a relatively narrow range in spite of wide fluctuations in the demand for nutrients and body’s supply (1). Circulating glucose is derived from three sources: intestinal absorption during the fed state, glycogenolysis and gluconeogenesis (2). After feeding, glucose plasma levels increase, and insulin is secreted from pancreatic β cells. This hormone increases the disposal of glucose by peripheral tissues, glycogen synthesis and lipogenesis in the liver (3), and promotes the uptake of glucose and conversion to glycogen or triglycerides by muscle and adipocytes, respectively (4, 5). Insulin also acts on the liver to inhibit glucose production through gluconeogenesis and glycogenolysis (6). These actions reduce glucose levels in blood. In contrast, during fasting state, insulin levels progressively decrease, and counterregulatory hormones (mainly glucagon, adrenaline, glucocorticoids and growth hormone) (7) are released to the bloodstream to boost glucose production. In this condition, the liver is the principal source of plasma glucose, as it is the principal organ for the storage of glucose in the form of glycogen and endogenous glucose production. During short-term fasting periods, the liver produces glucose mainly by glycogenolysis, which is the breakdown of glycogen to glucose. As glycogen is dwindling, gluconeogenesis, which is *de novo* glucose synthesis obtained from non-carbohydrate precursors (mainly lactate, glycerol and amino acids), becomes the main mechanism to maintain blood glucose levels (8). Together with the liver, kidneys and small intestine are also relevant gluconeogenic tissues in acidotic conditions and after prolonged fasting (9–12). A proper coordination of these hypo- and hyperglycemic mechanisms ensure the maintenance of glucose homeostasis. Type 2 diabetes develops when there is a disruption in this fine-tuned balance between glucose generation and consumption, causing elevated blood glucose levels, increasing the risk of diabetic retinopathy, kidney disease, diabetic neuropathy, and macrovascular complications (13, 14). Thus, a precise mechanism to sense blood glucose levels is required, to adapt cell physiology to fluctuations in nutrient intake to maintain glucose homeostasis.

The regulation of glucose homeostasis involves the coordinated actions of multiple tissues and organs, including the central nervous system, liver, muscle, adipose tissue, and pancreas; and take places on a variety of levels, comprising hormone secretion, gene transcription and posttranslational modifications (PTMs) (8). PTMs of proteins allows cells to respond swiftly to internal and external signals *via* direct and dynamic regulation of protein function, localization and interaction. Due to their indispensable roles in regulating essential cellular processes, PTMs such as acetylation, phosphorylation, methylation, and ubiquitination have gained great attention during the past decades (15). Besides the well-studied PTMs, there are many others whose physiological and pathophysiological function begin to be unraveled. In this regard, the PTM *O*-GlcNAcylation has gained wide attention for its capacity to serve as a nutrient sensor, since its precursor, UDP-GlcNAc, is positioned at the nexus of glucose-, amino acid-, fatty acid- and nucleotide-metabolism through the hexosamine biosynthetic pathway (HBP). In addition to being dependent on nutrient availability, *O*-GlcNAc signaling is tightly regulated by different forms of cellular stress (16). This review cannot summarize all the extensive work about *O*-GlcNAcylation of proteins and their regulation and role in multiple physiological scenarios (we refer interested readers to excellent reviews (17–20). Instead, we would like focus on recent findings that highlight the function of *O*-GlcNAcylation in the modulation of systemic glucose homeostasis and its involvement in the metabolic syndrome and type 2 diabetes.

## *O*-GlcNAcylation

**Figure 1:**
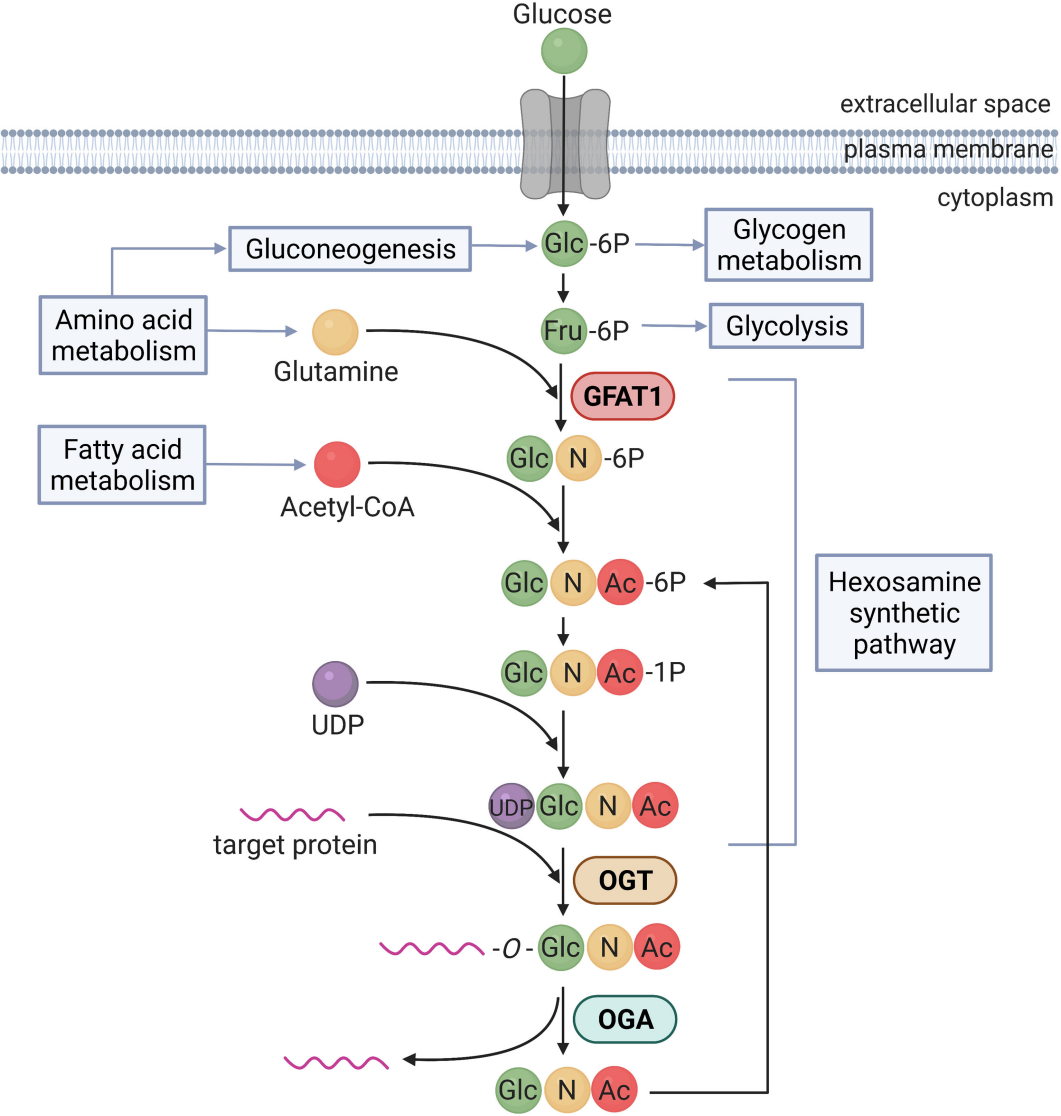
The process of protein modification by *O*-GlcNAcylation. This diagram illustrates the *O*-GlcNAcylation synthesis and salvage pathways, including the metabolic flux and regulation. Glucose (Glc) is incorporated to the cell and used for glycolysis, glycogen synthesis or hexosamine biosynthetic pathway. The enzyme Glutamine–fructose−6−phosphate amidotransferase 1 (GFAT1) converts fructose−6−phosphate (Fru−6P) in glucosamine−6−phosphate (GlcN−6P). Next, acetylation of GlcN−6P and uridylation of GlcN−1P take place, providing the donor substrate for protein *O*-GlcNAcylation named uridine diphosphateGlcNAc (UDP-GlcNAc). Then, the enzymes *O*-GlcNAc transferase (OGT) and *O*-GlcNAcase (OGA) catalyse the addition and removal of *O*-GlcNAc to the target protein, respectively. Free GlcNAc can be recycled through the generation of GlcNAc−6P, that can be utilized again by the hexosamine biosynthetic pathway. *O*-GlcNAcylation can be modulated at different points of the synthetic pathway by glucose, amino acids and fatty acids metabolism.

*O*-GlcNAcylation is an *O*-linked β-N-acetylglucosamine (*O*-GlcNAc) moiety linked to threonine and/or serine residues of proteins. Unlike canonical glycosylation, *O*-GlcNAcylation is not elongated to form complex structures, and can be localized mainly in nucleus, mitochondria, and cytoplasm (21). UDP-GlcNAc, the donor substrate for *O*-GlcNAcylation, is the end-product of HBP (**Figure 1**). The HBP is a branch of the glucose metabolic pathway, consuming approximately 2-5% of the total glucose (22). The synthesis of UDP-GlcNAc is controlled by a large number of metabolic pathways. Generation of fructose-6-phosphate by glycolysis represents the first step of the HBP to produce UDP-GlcNAc. Amino acid metabolism is also connected to HBP flux at several levels, mainly through glucose, gluconeogenesis and glutamine, the latter acting as nitrogen donor to build glucosamine-6-phosphate. Moreover, lipid metabolism is able to affect the HBP, *via* acetyl-CoA. UTP, which acts as donor to form UDP-GlcNAc, links flux through the HBP to both energy and nucleotide metabolism. Overall, UDP-GlcNAc represents a major hub integrating cellular metabolic pathways, being responsive to flux through them (23).

*O*-GlcNAcylation is a reversible and highly dynamic process, in a fashion analogous to phosphorylation: it rapidly cycles onto serine/threonine residues of target proteins and turns over more rapidly than the proteins which it modifies. Unlike phosphorylation, in which there are up to 500 kinases modulating human protein phosphorylation (24), *O*-GlcNAcylation is under the control of two enzymes: OGT, which catalyzes the transfer of a *O*-GlcNAc residue from the donor substrate UDP-GlcNAc to target proteins; and OGA, which catalyzes the hydrolysis of this sugar modification (18). It is also important to note that the glutamine:fructose-6-phosphate amidotransferase (GFAT), that catalyzes the conversion of fructose-6-phosphate to glucosamine-6-phosphate, plays as well a major role in the regulation of *O*-GlcNAcylation. As the first and rate-limiting enzyme in the HBP, GFAT activity is key in governing the availability of the end-product UDP-GlcNAc (25).

Maintaining homeostasis of *O*-GlcNAcylation is crucial for the normal function of multiple cellular processes that range from transcription and translation to signal transduction and metabolism (26), with a huge impact in glucose metabolism and insulin resistance. Hyperglycemia has been found to increase cellular *O*-GlcNAcylation in a number of tissues *in vivo* (27, 28). However, accumulating data revealed that the relationship between nutrient availability and *O*-GlcNAcylation cannot be reduced to a simple positive correlation, being elevated in many tissues during fasting. One potential mechanism that can explain this latter phenomenon is an increase in protein levels of OGT, which may induce the intracellular *O*-GlcNAcylation upregulation in spite of the limited availability of free UDP-GlcNAc (29–31) We will examine the effect of *O*-GlcNAcylation in the main organs and tissues involved in glucose metabolism and its effect on glucose homeostasis and type 2 diabetes development.

## Protein *O*-GlcNAcylation in the Hypothalamus

The brain plays a key role in the maintenance of glucose homeostasis, in particular homeostatic regions such as the hypothalamus. The hypothalamus is constituted by functionally and morphologically distinct nuclei, including the lateral (LHA), paraventricular (PVN), ventromedial (VMN), and arcuate (ARC) nuclei, which all contain specific glucose-sensing neuronal populations. The ARC contains two antagonist neuronal populations: anorexigenic neurons that release the pro-opiomelanocortin (POMC)-derived peptide, α-melanocyte-stimulating hormone (α-MSH), and orexigenic agouti-related peptide (AgRP)-producing neurons. Together with neurons expressing the melanocortin 4 receptor (MC4R), they constitute the melanocortin system, key for glucose metabolism (32). AgRP neurons also contain the orexigenic neuropeptide Y (NPY) (33). Previous studies demonstrated that *O*-GlcNAcylation is essential for optimal neuronal function and development (34), also that global *O*-GlcNAcylation and OGT levels are increased in response to glucose deprivation *in vitro* in neuronal cell lines (29). But the physiological relevance of *O*-GlcNAcylation in the hypothalamus and in glucose metabolism came later, with *in vivo* studies evaluating the role of OGT in different hypothalamic nuclei. Thereby, it was reported that mice subjected to food deprivation for 24 h or treated with the orexigenic hormone ghrelin, displayed higher levels of both global *O*-GlcNAc and OGT in AgRP neurons (35). As a result of the specific deletion of OGT in AgRP neurons, hepatic gluconeogenesis was attenuated, as indicated by reduced glucose levels during glucose and pyruvate tolerance tests, as well as reduced hepatic expression of gluconeogenic genes, without changes in circulating insulin levels or insulin sensitivity. When challenged with high fat diet, OGT ablation specifically in AgRP neurons exerted protective actions against diet-induced obesity and insulin resistance. Mechanistically, these effects were mediated at least in part by the promotion of WAT browning, leading to an amelioration of glucose metabolism.

An independent study demonstrated that, on the contrary, in the hypothalamic PVN, fasting reduced *O*-GlcNAcylation in αCaMKII-positive cells, while there was a marked upregulation of *O*-GlcNAc and OGT after incubation with glucose in a dose-dependent manner (36). Selectively OGT deletion in the αCaMKII cells of the PVN caused obesity and hyperphagia, as well as increased fat mass and liver weight gain (36, 37). Consequently, these mice showed a significant impairment in both glucose and insulin tolerance tests under standard diet fed conditions, and an exacerbated peripheral insulin resistance induced by high fat diet (38). While scarce *in vivo* data point that *O*-GlcNAcylation have a huge and nuclei-specific effect, it is important to note that to date, the possible role of *O*-GlcNAcylation in VMN neurons, which are essential to initiate the glucose counter-regulatory response to hypoglycemia, remains to be evaluated.

## Protein *O*-GlcNAcylation in the Pancreas

The pancreas is constituted by two different parts: the exocrine pancreas, which is a reservoir of digestive enzymes, and the endocrine islets of Langerhans, containing endocrine cells including α and β cells. β cells secrete insulin in response to high blood glucose, while α cells secrete glucagon in response to hypoglycemia. It was found that OGT is richly expressed in the pancreas (39), and it seems to be more abundant in islets in comparison to the acinar tissue (40). At functional level, it was recently described that *O*-GlcNAcylation in α cells was required for the glucagon secretion and for appropriate α cell function *in vivo* (41). Despite α cell-OGT KO mice display normal glucose tolerance and insulin sensitivity, when subjected to a pyruvate tolerance test, which evaluates the gluconeogenic capacity in a hypoglycemic condition, they exhibited significantly reduced blood glucose production compared with the control, indicating impaired gluconeogenesis. The underlying mechanism implies that OGT deflection in α cells caused an impairment in islet glucagon secretion and α cell content in young mice, and reduced mass of α cells in older mice (41).

In β cells, *O*-GlcNAc levels are sensitive to glucose (42), suggesting that *O*-GlcNAc could function as a glucose sensor regulating insulin secretion. In fact, it was described that *O*-GlcNAcylation was also key for function and survival of β cells, being indispensable for the control of both insulin secretion and β cell number. Specifically, standard diet-fed mice harboring OGT deletion in β cells exhibited severe hyperglycemia, glucose intolerance, impaired insulin secretion and developed severe diabetes due to β cell failure (40). OGT is also needed for coupling hyperlipidemia to β cell functional adaptation during the compensatory prediabetic phase, characterized by the hypersecretion of insulin to stabilize blood glucose and forestall diabetic progression (43). Thus, HFD feeding transiently upregulated β cell *O*-GlcNAcylation to potentiate insulin secretion. In line with these results, OGA overexpression in β cells decreased insulin gene expression, islet insulin content and blood insulin levels (44). Furthermore, OGT loss caused hyperproinsulinemia, because of a failure of proinsulin-to-insulin processing, resulting in hyperglycemia and the appearance of the same defects in islets that are found in patients with type 2 diabetes (45).

While *O*-GlcNAcylation seems to be essential for optimal β cell function under physiological and prediabetic conditions (46, 47), chronic hyperglycemia led to hyper-GlcNAcylation of several proteins, promoting impaired insulin secretion (44, 48, 49) and pancreas apoptosis (50).

## Protein *O*-GlcNAcylation in the Liver

Liver plays an essential part in the maintenance of glucose homeostasis, since it has the capacity to store excessive glucose as glycogen; and to produce new glucose from glycogenolysis and gluconeogenesis from non-carbohydrate precursors. Early *in vitro* assays have shown that *O*-GlcNAcylation of a limited number of proteins is elevated during nutrient deprivation, an effect driven by a significant increase in OGT protein levels (30). Interestingly, some of the targets of glucose deprivation-induced *O*-GlcNAcylation are different from those altered in response to high glucose treatment, suggesting that different proteins are specifically *O*-GlcNAcylated upon glucose deprivation or glucose excess. Later, it was found that one of the processes most regulated by *O*-GlcNAcylation during fasting is gluconeogenesis. In this sense, it has been reported that the master regulator peroxisome proliferator-activated receptor-gamma coactivator alpha (PGC1α) is *O*-GlcNAcylated, which facilitates its cooperation with OGT to target and modify forkhead Box O1 (FOXO1), an essential process to promote gluconeogenesis (51). A potential underling mechanism implies the activity of host cell factor 1 (HCF-1), which recruits OGT during nutrient deprivation to *O*-GlcNAcylate PGC1α, thus protecting it from degradation and promoting gluconeogenesis (52). The relevance of OGT as a mediator of gluconeogenesis was given by the fact that the OGT knockdown in hepatocytes, *in vivo* and *in vitro*, significantly suppressed the induction of gluconeogenic genes, pyruvate-induced and fasting-induced gluconeogenesis (31, 52). OGT and *O*-GlcNAcylation have also emerged as important mediators of the glucose counterregulatory response, being upregulated by glucagon (31, 53), adrenaline and cortisol (31). In a recent study, it was found that the tumor suppressor p53 was an essential transcription factor for PCK1 to boost hepatic glucose production during nutrient deprivation, and that the gluconeogenic function of p53 was mediated by *O*-GlcNAcylation (31). Specifically, p53 was stabilized by *O*-GlcNAcylation in serine 149 during nutrient deprivation, and mutant p53S149A failed to promote gluconeogenesis. Moreover, the genes responsible for *O*-GlcNAcylation were increased in the liver of patients with type 2 diabetes, being positively correlated with high glucose during fasting and HOMA-IR. In the same line, and in an independent study, it was also described that OGT overexpression promoted insulin resistance (54), and that hyperglycemia promotes the *O*-GlcNAcylation of CREB-regulated transcription coactivator 2 (CRTC2), which induces the transcription of gluconeogenic genes, contributing to increase hepatic glucose production in diabetic animal models. Supporting these results, decreasing amounts of *O*-GlcNAcylated CRTC2 by the overexpression of OGA lowered the gluconeogenic profile (28).

Collectively, *O*-GlcNAcylation of transcription factors and cofactors involved in gluconeogenesis stimulates hepatic glucose production under physiological and pathophysiological conditions, suggesting that hepatic *O*-GlcNAc signaling deserves great attention to further understand the development of T2D.

## Protein *O*-GlcNAcylation in the Adipose Tissue

As insulin-sensing tissues, both brown (BAT) and white adipose tissue (WAT) play a crucial role in glucose homeostasis and energy balance. BAT plays a key role in thermogenesis, and for this function, it requires the usage of fatty acids and glucose (55, 56). Contrary, WAT has the capacity to storage lipids and release them when necessary due to energy requirements. During acute fasting, high levels of *O*-GlcNAcylation in visceral adipose tissue retained its fat mass. Loss of OGT specifically in white adipocytes promoted lipolysis in visceral fat by decreasing *O*-GlcNAcylation, while adipose OGT overexpression diminished lipolysis in the adipose tissue and promoted diet-induced obesity, glucose intolerance and insulin resistance (57). In line with this observation, previous *in vitro* studies in 3T3-L1 adipocytes have shown that elevated *O*-GlcNAcylation levels led to insulin resistance (58). *In vivo*, this *O*-GlcNAcylation-induced insulin resistance is mediated, at least in part, by the interaction between phosphatidylinositol 3,4,5-trisphosphate and OGT, which results in the translocation of OGT from the nucleus to the plasma membrane, where the enzyme catalyses dynamic medication of the insulin signaling pathway, including AKT, by *O*-GlcNAc, attenuating insulin signal transduction (54, 59). Elevated *O*-GlcNAcylation also contributed to insulin resistance in adipocytes by modifying IRS-1 and inhibiting its phosphorylation, impairing insulin signaling (60). In an independent study, it was demonstrated that OGT ablation in white adipocytes of mice fed with HFD protected against diet-induced obesity and improves glycemic control. Specifically, OGT deletion reduced circulating insulin levels and improved HOMA-IR, as well as glucose tolerance and insulin sensitivity. This general improvement was also found in the liver, which showed reduced lipid content (61).

*O*-GlcNAcylation in BAT has also been shown to play a relevant role in thermogenesis and glucose homeostasis (62). OGT deletion in brown adipocytes led to decreased mitochondrial expression and PGC1α levels. Consequently, these mice are unable to maintain blood glucose levels during cold exposure, impairing cold-induced thermogenesis (63, 64). Not only *O*-GlcNAcylation become essential for proper BAT function, but also for browning of subcutaneous white adipose tissue, protecting mice form high-fat diet-induced obesity and hepatic steatosis, improving glucose metabolism (65).

## Protein *O*-GlcNAcylation in the Skeletal Muscle

Skeletal muscle is the largest insulin-sensitive tissue and is the responsible for more than the 80% of insulin-stimulated glucose uptake in humans (66). *In vitro* studies showed that in skeletal muscle, a global upregulation of *O*-GlcNAcylation generated insulin resistance since it inhibited glucose uptake (64, 67). This phenomenon was consistently observed *in vivo* in both mice and human patients (68). Global *O*-GlcNAcylation levels were found augmented in type 2 diabetic patients, and OGT deletion specifically in skeletal muscle in mice improved insulin sensitivity and increased GLUT4 protein levels in gastrocnemius muscle of the KO mice in comparison to control mice. In addition, mice lacking OGT in skeletal muscle were protected against HFD-induced insulin resistance. Consistently, after being fed for 12 or 22 weeks with HFD, mice lacking OGT in skeletal muscle displayed lower blood glucose during fasting, and improved insulin sensitivity and glycemic control (68).

## Discussion and Remarks

**Figure 2:**
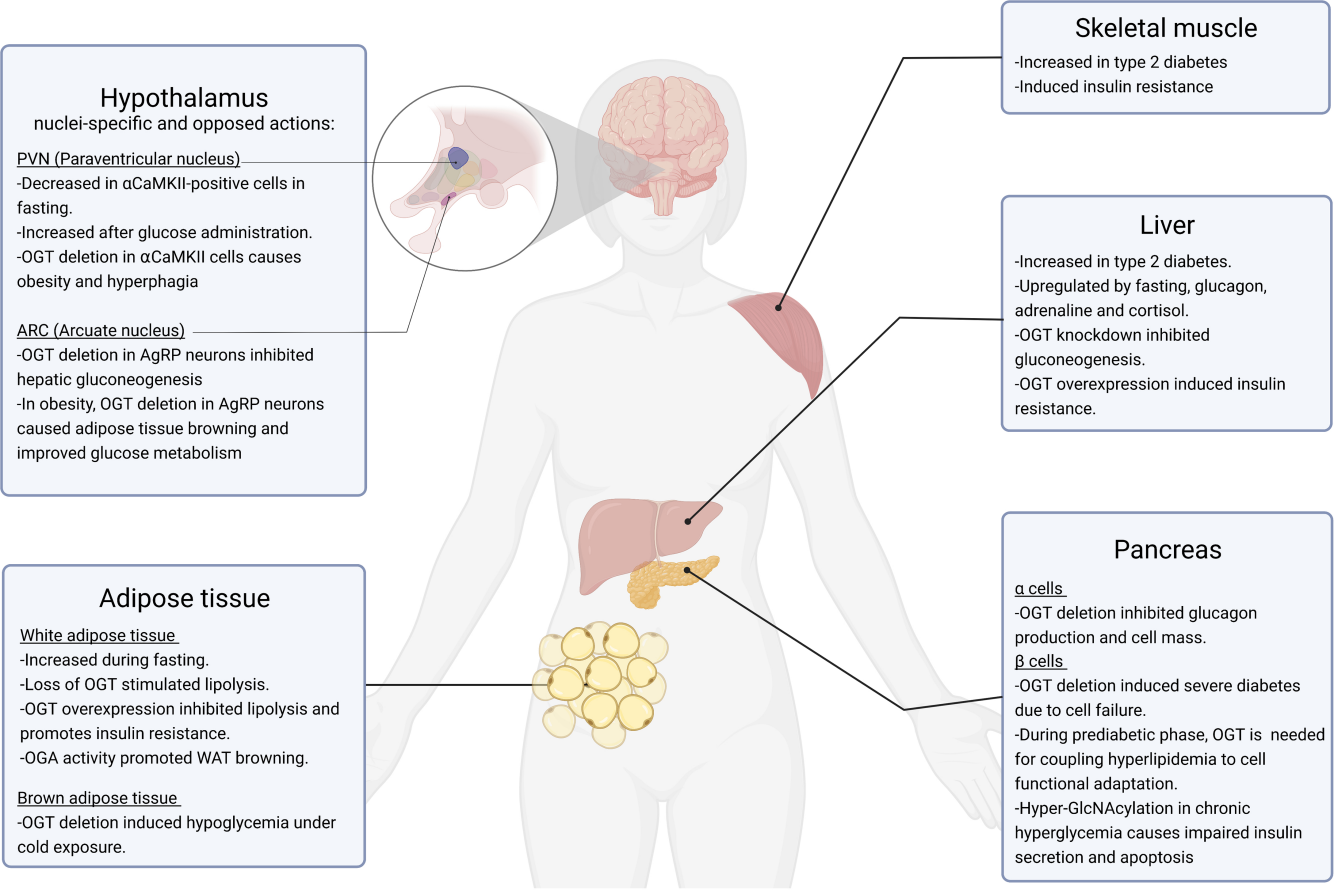
Role of *O*-GlcNAcylation in glucose homeostasis. This figure summarizes the currently known physiological and pathophysiological roles of *O*-GlcNAcylation in organs involved in the regulation of glucose homeostasis, including the hypothalamus, white and brown adipose tissue, skeletal muscle, liver and pancreas. All these tissues are affected by type 2 diabetes, obesity and related metabolic states.

The cellular signaling machinery is a complex network of components which is only partially understood. In the past years, *O*-GlcNAcylation has emerged as an effective toolbox to tune and integrate signaling pathways in fundamental cellular processes, such as glucose homeostasis (**Figure 2**). As different studies report, *O*-GlcNAcylation is finely regulated by nutrient availability in a tissue-dependent manner, being active in every major tissue involved in the regulation of systemic glucose homeostasis. While physiological *O*-GlcNAcylation is essential to maintain proper cellular functions, nutritional stress and hyperglycemia induce *O*-GlcNAcylation of some key transcription factors and cofactors, which in turn further increases glucose levels, generating a vicious cycle that worsens the glucose toxicity and aggravates the progression of diabetes and diabetic-related complications.

The advances in the understanding of *O*-GlcNAcylation under both homeostatic and type 2 diabetes during the last years are undeniable. However, different questions regarding the role of *O*-GlcNAcylation in the pathological condition remain unanswered. Among others, little is known about the role of this posttranslational modification in diabetes-related diseases such as fatty liver, sarcopenia and cardiovascular failure. Furthermore, a better characterization of *O*-GlcNAcylation target proteins is required, as well as its relation with other posttranslational modifications, making special emphasis on master metabolic regulators of glucose metabolism. In the future, it will be necessary to determine the fine tune regulation of specific individual *O*-GlcNAcylated residues of target proteins under different homeostatic and pathological conditions. Additionally, it will be essential to determine the impact of these individual moieties on the stability, dynamics and enzymatic activity of target proteins, as well as their relevance in the overall metabolism. Unraveling these phenomena will not only lead to understand the molecular mechanisms behind pathological development, but will also offer novel ways to diagnose and develop effective therapies to diabetes and diabetic complications.

## Author Contributions

MG-R, MF, CD, and RN contributed to the conception and design of the article and interpreting the relevant literature. All authors reviewed and approved the final version of the manuscript.

## Funding

This work has been supported by grants from FEDER/Ministerio de Ciencia, Innovación y Universidades-Agencia Estatal de Investigación (CD: BFU2017-87721; RN: RTI2018-099413-B-I00 and RED2018-102379-T), Xunta de Galicia (RN: 2015-CP080 and 2016-PG057), Fundación BBVA (RN), Fundación Atresmedia (RN), European Foundation for the Study of Diabetes (RN). This research also received funding from the European Community’s H2020 Framework Programme (ERC Synergy Grant-2019-WATCH- 810331, to RN). CIBER de Fisiopatología de la Obesidad y Nutrición (CIBERobn) is an initiative of the Instituto de Salud Carlos III (ISCIII) of Spain which is supported by FEDER funds.

## Conflict of Interest

The authors declare that the research was conducted in the absence of any commercial or financial relationships that could be construed as a potential conflict of interest.

## Publisher’s Note

All claims expressed in this article are solely those of the authors and do not necessarily represent those of their affiliated organizations, or those of the publisher, the editors and the reviewers. Any product that may be evaluated in this article, or claim that may be made by its manufacturer, is not guaranteed or endorsed by the publisher.
